# Severe acute respiratory infections: An epidemiological analysis of surveillance data in Bahrain, 2018–2022

**DOI:** 10.5339/qmj.2024.25

**Published:** 2024-07-01

**Authors:** Adel Al Sayyad, Afaf Merza Mohamed, Bayan Abduljalil Alajaimi, Ebrahim Matar, Wafa Fawzi Hasan, Qatmeer Aldolabi, Abdulla Khalaf Abdulla Abdulmahdi, Mohamed Saeed Yusuf

**Affiliations:** 1 Epidemiology & Public Health, Chief of Disease Control Section, Ministry of Health, CMMS, AGU, Manama, Bahrain; 2 Public Health Directorate, Ministry of Health, Manama, Bahrain *Email: Ematar1@health.gov.bh

**Keywords:** Influenza, respiratory syncytial virus, epidemiology, severe acute respiratory infections, Bahrain

## Abstract

**Background:**

Severe acute respiratory tract infections (SARI) pose a health threat to children and adults worldwide. The SARI surveillance program was initiated in 2018 in Bahrain to monitor the activity of respiratory pathogens. Salmaniya Medical Complex (SMC) was chosen as the sentinel site for the SARI surveillance program. This study aimed to describe the epidemiology of SARI patients admitted to SMC from 2018 to 2022.

**Methods:**

Patients meeting the World Health Organization definition of SARI and presenting with cough and fever within the last 10 days and admitted to SMC from January 2018 until December 2022 were included in the study. Epidemiological data on SARI cases were collected from SARI surveillance data and analyzed using SPSS version 25 and Excel.

**Results:**

A total of 1362 SARI cases were enrolled from January 2018 to the end of December 2022; the majority were males (57.7%, *n = *786). The highest SARI incidence rates were recorded among individuals over 65 years old (155.5 per 100,000) in 2021 and among those under 5 years old (887 per 100,000) in 2020. About half of the patients had at least one comorbidity (54.0%, *n = *735), with diabetes (23.0%, *n = *313) and hypertension (17.2%, *n = *234) being the most common. The highest number of cases was observed in 2021 (27%, *n = *373), followed by 2018 (20%, *n = *267). A viral pathogen was detected in 30.7% (*n = *418) of the SARI patients. The most prevalent pathogen was influenza A (11.5%, *n = *156), followed by SARS-CoV-2 (9.7%, *n = *132), respiratory syncytial virus (RSV) (5.1%, *n = *69), and influenza B (3.9%, *n = *53). The highest percentage of SARI cases was recorded in the winter months, mainly January (17%, *n = *236). The percentages of influenza A and RSV cases were highest in December, at 22% (*n = *39) and 14% (*n = *25), respectively. Influenza B cases were recorded predominantly in March (9%, *n = *11).

**Conclusion:**

The incidence of SARI was highest among patients above 65 years old. The majority had comorbidities. Influenza and respiratory syncytial viruses were the most frequent causes of SARI, with influenza A being the most prevalent. December and January were the months with the highest SARI cases and viral detection rates. Promoting vaccination, timely testing, and prompt treatment, especially for the elderly and those with comorbidities, is key to reducing SARI-related morbidity and mortality, especially during peak seasons.

## Introduction

Influenza refers to both a viral respiratory illness that is characterized by fever, malaise, and myalgia and the causative single-strand negative sense RNA orthomyxoviruses. Influenza viruses include types A, B, C, and D. Influenza A and B are the most known to cause seasonal epidemics.^1^ Further influenza A is known to cause some of the deadliest pandemics in human history, which is attributed to the genetic variation occurring each season. In 1918, the Spanish flu caused by the H1N1 virus resulted in about 50 million deaths, 20 million also died due to the H2N2 influenza virus (Asian flu) in the 1950s, and the H3N2 influenza (Hong Kong flu) in the 1960s.^2-4^ In 2009, the estimated number of deaths during the H1N1 pandemic ranged from 150,000 to 575,000 deaths.^5^

The term “influenza” is also broadly used to describe any viral respiratory illness.^6^ It is important to distinguish influenza from other viral respiratory infections due to its systemic symptoms, its tendency to cause winter epidemics, and its ability to spread quickly among close contacts.^7^ Influenza viruses circulate around the world, causing seasonal influenza outbreaks.

The threat of acute respiratory tract infections is one of the largest in the world. It is estimated that influenza viruses in the United States alone cause 12,000–61,000 deaths each year.^8^ Globally, influenza viruses are estimated to cause 291,000–645,000 deaths annually.^9^

The incubation period of influenza viruses ranges from 1 to 4 days,^10^ with a serial interval of 3–5 days.^11^ A characteristically uncomplicated influenza illness starts with the acute onset of fever, cough, and body pain.^12^ Depending on the virus type and the patient’s characteristics, a complicated illness and poor outcomes could occur.

Hence, influenza surveillance programs are needed to provide valuable information to public health authorities and policymakers to inform appropriate prevention and control strategies. Since 1952, the Global Influenza Surveillance and Response System (GISRS) has been monitoring the circulation of influenza viruses. In 2011, the World Health Organization adopted the new Pandemic Influenza Preparedness Framework.^13^

Influenza surveillance programs are used to create baseline data on virus activity, detect locally circulating virus strains, identify the seasonality of viruses, estimate the disease burden, evaluate the effectiveness of vaccines and antivirals, and spot unusual events that may herald novel viruses.

Global public health relies heavily on severe acute respiratory tract infections (SARI) surveillance to monitor and respond to severe respiratory infections. The process involves integrating surveillance systems, standardized case definitions, and laboratory diagnostics in order to detect and track outbreaks of respiratory diseases. Global collaboration and data sharing through global networks such as the GISRS enable early detection of pathogens and establish the basis for health interventions. SARI surveillance data are utilized to estimate disease burden and severity, lead vaccine development, and outline health policies. Through an enhanced understanding of respiratory disease trends and coordinated responses, SARI surveillance contributes to global respiratory health prevention and control.^14^ Further SARI surveillance programs generate baseline data to which emerging pandemics could be compared.^15^

Bahrain is a small archipelago with about 1.5 million inhabitants.^15^ In Bahrain, the SARI surveillance program was updated in 2018 to be aligned with the recent WHO recommendations. The provision of secondary and tertiary care in Bahrain is carried out through three governmental hospitals: Salmaniya Medical Complex (SMC), Bahrain Defense Force (BDF) Hospital, and King Hamad University Hospital (KHUH). Both BDF and KHUH provide care mainly to the Ministry of Defense and Interior personnel and their families.^16^ SMC, being the main and largest secondary hospital in Bahrain, was selected as the sentinel surveillance site.^17^ SMC provides emergency, outpatient, secondary, and tertiary care to the entire population of Bahrain, making it the most representative site for effective surveillance.^18^

Several studies documented the epidemiological and virological variance in SARI patients, between influenza seasons. A study conducted in Central America found that the most common viruses circulating were respiratory syncytial virus (RSV) and influenza A virus. All cases of viral co-infection occurred in children less than 5 years old. RSV and influenza virus were respectively identified mainly during July–November and May–July.^19^ In Congo, influenza A virus was the most common virus; the majority of cases were children under 5e years old and occurred during the rainy season.^19^ In the Eastern Mediterranean Region, influenza A (H1N1) pdm09 and RSV were found to be the most common respiratory infections circulating, with most cases also occurring in children under 5 years old.^20^

There were no published studies that described the epidemiology, distribution, and seasonality of influenza viruses among SARI patients in the Kingdom of Bahrain. This study aims to describe the epidemiology, distribution, and seasonality of circulating viruses among SARI patients admitted to a sentinel site in Bahrain from 2018 to 2022.

## Methods

### Study design and study setting

This is an epidemiological analysis of collected SARI surveillance data from January 2018 through December 2022 in the Kingdom of Bahrain. The SARI surveillance program was updated in 2018 to adopt the latest definitions and measures recommended by the WHO SARI surveillance program, and SMC was chosen as the sentinel site for surveillance. Epidemiological and virological data were collected on a daily basis from SARI patients admitted to the SMC. Data were then uploaded to the WHO Eastern Mediterranean Flu Network (EMFLU) website.

### Study population

SARI is defined by the WHO as any patient with an acute respiratory infection, presenting with cough, a history of fever, or a measured temperature ≥38°C in the last 10 days that required hospital admission.^21^ This definition was used in the SARI surveillance program conducted in Bahrain. All patients who met the SARI definition and were admitted to SMC from January 2018 to December 2022 were included in the study.

### Data collection

An assigned public health specialist from the public health directorate was responsible for collecting the data from patients fitting the SARI criteria. Collected data included: patient’s age, sex, address, influenza vaccination status, symptoms onset date, reporting date, date of collection of nasopharyngeal specimens, reverse transcription-polymerase chain reaction (RT-PCR) for influenza test result, date of hospital admission, date of discharge, outcome status as dead or alive, intensive care unit (ICU) admission during the stay, the requirement of mechanical ventilation, administration of antiviral medication, and medical comorbidities. Demographic data of Bahrain were obtained from Bahrain’s Open Data Portal.^22^

### Ethical approval

The study adhered to the Helsinki Declaration guidelines and received ethical approval from the Ministry of Health’s Health Research Committee in the Kingdom of Bahrain (AUPH-2024-00060). The data, anonymized prior to receipt, were obtained by the research team from the influenza surveillance team.

### Specimen collection and testing

All enrolled patients fitting the SARI criteria underwent RT-PCR tests to identify the causative agent. The specimens were collected via nasopharyngeal swabs and then refrigerated in viral transport media until processed within 24 h at the National Influenza Center, where they underwent testing for influenza viruses (A and B), respiratory syncytial virus, adenovirus, and SARS-CoV-2 using the Fast Track Diagnosis Respiratory Pathogens 21 Assay.^23^ All specimens were collected, transported, and processed in accordance with the WHO standards as outlined in the “Manual for the Laboratory Diagnosis and Virological Surveillance of Influenza.”^24^

### Statistical analysis

The data for the patients were initially retrieved from the EMFLU database. The refined data were then analyzed in SPSS version 25, which enabled in-depth analyses of rates, trends, patterns, and relationships, revealing insights into patient characteristics and outcomes. Additionally, Microsoft Excel was utilized as a complementary tool to create clear and informative visualizations. The mean and standard deviations were reported for the quantitative variables, whereas the qualitative variables were summarized with counts and percentages. Incidence rates specific to different age groups were computed using mid-year population statistics.

## Results

### Total SARI

A total of 1,362 patients were registered during the surveillance period between 2018 and 2022. The years of highest patient enrollment were 2019 and 2021, with 359 (26.3%) and 373 (27.4%) patients, respectively (Table 1).

**Figure 1.**
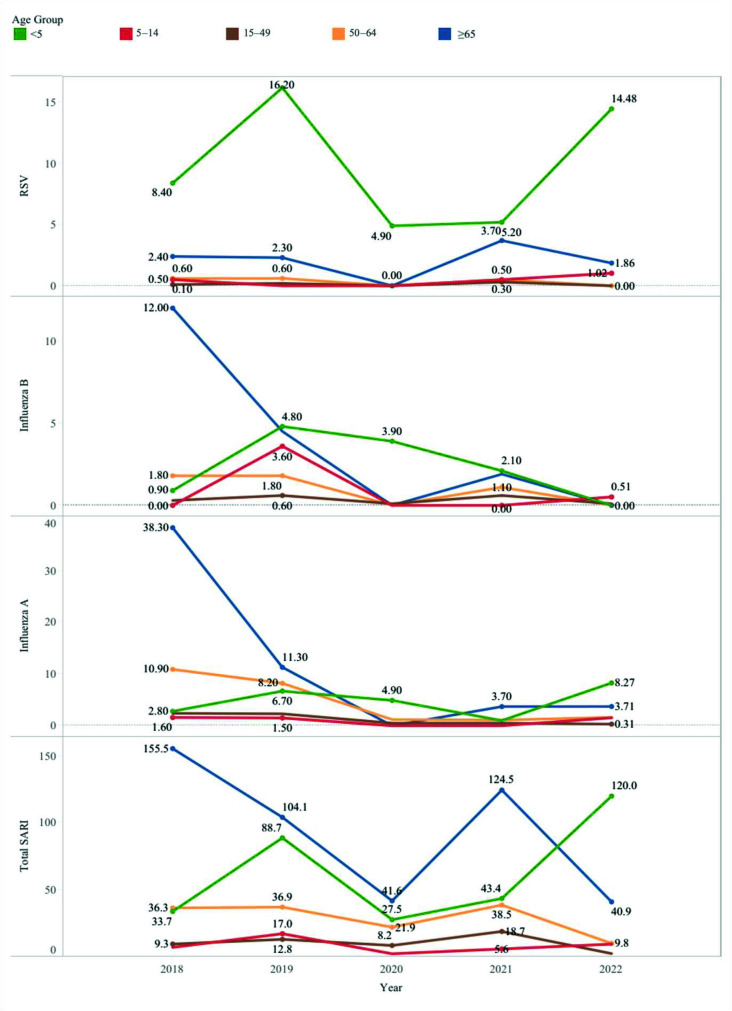
Age-specific incidence rates of SARI cases per 100,000 from 2018 to 2022 in the Kingdom of Bahrain.

Male patients constituted most of the study population: 786 patients (57.7%). The average overall incidence of SARI was highest among patients aged above 65 years and those aged below 5 years, with incidences of 93 and 63 cases per 100,000 individuals, respectively (Figure 1).

Viral pathogens were detected in 418 (30.7%) patients. Among the identified viruses, influenza A was the most prevalent with 156 patients (37.3%) (Table 1).

Over half of the patients presented with at least one comorbidity 735 (54%), and 346 (25%) had two or more comorbidities. The most frequently observed diseases were diabetes in 313 patients (25%) and heart disease in 234 patients (18%) (Table 1).

Among the enrolled patients, 199 (14.6%) patients were admitted to the ICU, 124 (9%) required mechanical ventilation, and 87 (6.3%) patients died (Table 1). A seasonal trend in SARI cases was evident, with the highest number of cases recorded between November and February, peaking in January with 236 (17.3%) patients. The lowest cases were recorded in May and June, with 52 (3.8%) and 53 (3.9%) cases, respectively.

### Influenza A

During the 5-year surveillance period, 11.45% (156) of SARI cases were diagnosed with influenza A. The highest prevalence of influenza A cases was recorded in 2018, with 41% (64) of the patients testing positive, followed by 32.7% (51) in 2019. Subsequent years showed a decline in the number of cases, reaching its lowest point in 2021 with only 6.4% (10) cases.

Regarding the gender distribution, 59% (92) of the patients who tested positive were males (Table 1).

During the surveillance period, the average incidence rate of influenza A was highest among individuals over 65 years old, at 11 cases per 100,000, and lowest in the age group between 5 and 50 years, with approximately 1 case per 100,000 individuals. In the years 2018 and 2019, influenza A primarily affected the above 65-year-old population, reaching an incidence rate of 38 cases per 100,000 individuals in 2018. Despite remaining the most affected group in 2019, the incidence rate decreased to 11 cases per 100,000. This age group was closely followed by the 50- to 65-year-old group, which recorded an incidence rate of 8.2 cases per 100,000. In 2020 and 2022, the incidence of influenza A was the highest in children below 5 years old, approximating 5 and 8 cases per 100,000 individuals, respectively (Figure 1).

**Figure 2.**
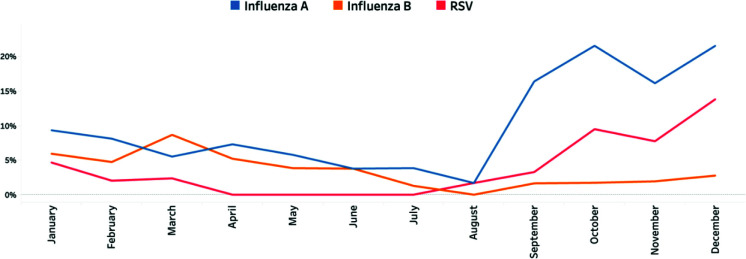
The percentage of positive SARI cases in the period from 2018 to 2022 in the Kingdom of Bahrain.

In relation to influenza A seasonality, the percentage of positive influenza A samples peaked between October and December, with 22% (25), 16% (25), and 22% (39), respectively. The lowest percentages were recorded between June and August, with respective percentages of 4% (2), 4% (3), and 2% (1) (Figure 2).

Of the influenza A positive cases, the majority of the samples were un-subtyped (50.6%, 70), with H1N1 strains constituting 44.2% (69) and H3N2 strains making up 5.1% (8) of the cases. With respect to clinical outcomes, 21.8% (34) of the patients were admitted to the ICU, 11.5% (18) required mechanical ventilation, and mortality was observed in 7.7% (12) of influenza A SARI patients. Comorbidities were present in 57.7% (90) of the affected patients, with chronic heart disease and diabetes being the most prevalent at 20.5% (32) and 19.2% (30), respectively (Table 1).

### Influenza B

Fifty-three patients were diagnosed with influenza B, constituting 3.9% of all SARI cases. The reported number of influenza B cases varied across the observed years. An initial increase was observed from 12 cases (22.6%) in 2018 to 23 cases (43.4%) in 2019. The year 2020 saw a significant decline to 5 cases (9.4%), with a moderate rise to 11 cases (20.7%) in 2021. The year 2022, however, marked the lowest number of cases, with only 2 cases (3.7%). The gender distribution among influenza B patients was equitably balanced (Table 1).

Analysis based on age groups revealed that the average incidence rate was notably higher at the extremes of age, with patients above 65 years and those below 5 years accounting for 3.7 and 2.3 cases per 100,000, respectively. In 2018, the incidence rate peaked in those aged above 65 years, with 12 cases per 100,000. The year 2019 saw an equivalent incidence in patients above 65 years old and those less than 5 years old, with rates of 4.5 and 4.8, respectively. In 2020, the incidence rate for patients under 5 years old remained relatively constant at approximately four cases per 100,000, while no cases were reported among those above 65 years old. In 2022, the incidence rate approached zero across all age groups (Figure 1).

Seasonal trends in influenza B demonstrated peaks in January and March, with the percentages of positive samples reaching 6% (14) and 9% (11), respectively (Figure 2).

Outcomes for influenza B in SARI patients indicated that 13.2% (7) were admitted to the ICU, 3.8% (2) required mechanical ventilation, and a mortality rate of 5.6% (3) was recorded. A proportion of 43% (23) of influenza B patients presented with comorbidities, with asthma emerging as the most prevalent associated condition (Table 1).

### RSV

In the observational period, 68 cases of RSV, accounting for 5% of total SARI cases, were reported. The year 2018 witnessed 19% (13) of RSV cases, which increased to 30.8% (21) of the cases in 2019, marking the highest occurrence during the surveillance period. Subsequently, the cases decreased to a 5-year low in 2020, recording 7.3% (5) of the total cases. However, in 2021 and 2022, the numbers began to increase, reporting 17.6% (12) and 25% (17), respectively. Among the RSV patients, most were males, accounting for 57.3% (39) (Table 1).

The average incidence of RSV was highest among children below 5 years old, approximating 10 cases per 100,000 individuals. Specifically, the incidence among children under 5 was 8 cases per 100,000 in 2018, increasing to 16 cases per 100,000 in 2019. A decline was noticed in 2020 and 2021, with the rate dropping to around 5 cases per 100,000. However, in 2022, the incidence rate picked up to reach 14 cases per 100,000 (Figure 1).

Assessing the seasonality of RSV, the percentage of positive samples peaked between October and December, with proportions of 9% (11), 8% (12), and 14% (25), respectively. Conversely, the period from April to July recorded the lowest number of cases, with no RSV cases detected (Figure 2).

Regarding comorbidities, the majority of RSV patients did not exhibit any underlying diseases, with only 27.9% (19) having associated conditions. Asthma was the most prevalent comorbidity, accounting for eight of these cases. Concerning patient outcomes, 17.9% (12) were admitted to the ICU, the same percentage required mechanical ventilation, and a mortality rate of 6% (4) was recorded (Table 1).

## Discussion

SARI poses a huge burden on healthcare systems globally, with influenza viruses causing the most severe infections. The novel H1N1 influenza virus in 2009 sparked a pandemic that touched 74 nations on 6 continents and killed hundreds of thousands of people.^25^

A thorough understanding of the seasonality of influenza viruses is the first step in being prepared for the prevention and management of influenza outbreaks as well as unforeseen pandemics. Additionally, the detection of circulating influenza viruses aids vaccine producers in determining the components of the seasonal influenza vaccine. Furthermore, SARI surveillance enhances the understanding of influenza seasonality in our region and aids in optimizing vaccination timing to avoid significant outbreaks.^26^

No studies have examined SARI’s patterns, trends, and seasonality in Bahrain. This study is the first to describe the epidemiology of SARI among individuals in Bahrain. Between 2018 and 2022, 1,362 patients with SARI were enrolled in the study. Among the study population, males constituted the majority. The highest incidence of SARI was observed in patients over the age of 65 years and in children under the age of 5 years. Thirty percent of patients tested positive for viral pathogens, with influenza A and SARS-CoV-2 most prevalent (37.3% and 31.5%, respectively). Over half of the patients had at least one comorbidity, with diabetes and heart disease being the most frequently observed. Notably, 14.6% of patients were admitted to the ICU, 9% required mechanical ventilation, and 6.3% died.

A seasonal trend in SARI cases was evident, as the highest percentage of SARI cases were recorded in the winter months. This peak of SARI was driven mainly by influenza A and RSV cases which increased in December. While influenza B cases were recorded predominantly in March. This is in line with findings from both regional and global studies. A study conducted in the Eastern Mediterranean Region, also found that SARI cases surged in winter.^27^ An epidemiological study in Egypt also confirms that their influenza season peaks during the winter months.^28^ Furthermore, a study in Tunisia reported a peak in SARI cases among ICU patients during the winter.^29^ Similarly, studies conducted in Vietnam and Shanghai reported a surge in SARI cases caused by influenza during the winter season.^30,31^ However, in South America, the patterns exhibited some variations. A study in Chile recorded a peak in SARI cases during the winter (March–May) and autumn (June–August) months, while a study from Suriname did not identify any seasonal pattern for the occurrence of SARI cases.^32,33^

The seasonality of SARIs observed in the 2018–2022 period in Bahrain was similar to patterns found in other studies, where influenza infections are commonly seen during the winter season which includes the months of November, February, and January in the northern hemisphere.^28,34-36^ It is more likely that people will spend more time indoors during winter, which can lead to respiratory illnesses spreading more easily.^28^

Our study found that the incidence of SARIs was highest in children under the age of 5 years and in individuals over the age of 65 years, which is consistent with a study carried out in Pakistan.^37^ Studies conducted in Arizona and Chile revealed that ages above 65 years were the most affected.^32,38^ In contrast, the study conducted in Egypt found that children under 5 years of age were the most frequently admitted for SARI.^28^ Another study in Morocco found that most SARI patients were below the age of 15 years.^39^ Due to their increased susceptibility to influenza viruses, children suffer from more severe cases of influenza than other age groups.^40^ However, it is possible that parents’ commitment to seek care for their children may justify the higher number of children less than 5 years old at the SARI sentinel site in Bahrain. Additionally, unlike young children, adults can generally take over-the-counter medications instead of visiting a hospital for treatment.

The study had some limitations, as it was based on surveillance data. One of which was underreporting, where not every case fitting the definition is reported in a timely manner. Another is the lack of comprehensive data, where not all aspects of SARI are collected, such as presenting symptoms. While influenza A predominated during the surveillance period, most of the samples were reported without the subtype, as the subtype is usually done at a later stage. The surveillance period included the period of the COVID-19 pandemic, which laid an unprecedented strain on health care systems including that of the Kingdom of Bahrain. This may lead to a decline in the accuracy and quality of the reported data.

Nonetheless, this study has several strengths. It is the first study to describe the epidemiology of SARI in patients from Bahrain, encompassing 5 years of surveillance data. In our study, viral pathogens were assessed in each patient reported in the surveillance program via RT-PCR. Furthermore, selecting SMC as the sentinel site for Bahrain’s surveillance program carries substantial implications for the generalizability of the study’s findings. Given Bahrain’s small population of about 1.5 million and SMC as the main secondary hospital, admitting all age groups across all four governorates enhances the study’s relevance and generalizability of the results to Bahrain and similar settings.^17,18,41^

## Conclusion

From 2018 to 2022, the surveillance data showed a significant increase in the incidence of SARI among individuals aged over 65 years and those with comorbidities. Influenza A and RSV were the primary detected causative agents in SARI cases, with influenza A being the predominant pathogen identified. A seasonal pattern was observed in the occurrence of SARI, with the winter months of December and January being the peak months.

These findings highlight the importance of vaccination, timely testing, and prompt treatment of patients presenting with symptoms suggestive of influenza, especially for the elderly, and people with comorbid conditions, given their heightened risk of severe outcomes. Furthermore, intensifying vaccine promotion efforts before the peak of the influenza season is essential to boost immunity in the vulnerable, thus reducing infection and hospitalization rates.

## Authors’ Contributions

AA and AMM were the main contributors to the study design, literature review, data analysis, drafting of the manuscript, oversight for all phases of the project, and final approval of the version to be published. BAA, EM, and WHF were responsible for the literature review, data collection, data analysis, and drafting of the manuscript. QA was responsible for the data collection and revision of the manuscript. AKAA and MSY were responsible for the literature review and drafting of the manuscript. BA and EM were responsible for editing and reviewing the final manuscript. All the authors have read and approved the final manuscript.

## Acknowledgments

The authors gratefully acknowledge all medical staff working at the Pediatric and Medical departments at Salmaniya Medical Complex, the Public Health Laboratory Personnel in the National Influenza Center, and the Communicable Disease Surveillance Group in the Public Health Directorate.

## Disclaimers

The views expressed in the submitted article are our own and not the official position of any institution or funder.

## Conflict of Interest Statement

No financial or non-financial benefits have been received or will be received from any party directly or indirectly related to this article’s subject.

## Data Availability Statement

Data will be made available upon reasonable request.

## Ethical Approval

This study was conducted in accordance with the principles of the Helsinki Declaration, and it was ethically approved by the Health Research Committee in the Ministry of Health, Kingdom of Bahrain.

## Consent Form

Data received by the research team were anonymized.

## Figures and Tables

**Table 1.** SARI patients’ demographics, outcomes, and comorbidities by lab results for the period from 2018 to 2022 in the Kingdom of Bahrain.

		**Flu A**		**Flu B**		**RSV**		Others*		**Total**	
		**Count**	**%**	**Count**	**%**	**Count**	**%**	**Count**	**%**	**Count**	**%**
	2018	64	41.0%	12	22.6%	13	19.1%	178	16.4%	**267**	**19.6%**
	2019	51	32.7%	23	43.4%	21	30.9%	264	24.3%	**359**	**26.4%**
Year	2020	12	7.7%	5	9.4%	5	7.4%	146	13.5%	**168**	**12.3%**
	2021	10	6.4%	11	20.8%	12	17.6%	340	31.3%	**373**	**27.4%**
	2022	19	12.2%	2	3.8%	17	25.0%	157	14.5%	**195**	**14.3%**
	**Total**	**156**	**100.0%**	**53**	**100.0%**	**68**	**100.0%**	**1085**	**100.0%**	**1362**	**100.0%**
	Female	64	41.0%	27	50.9%	29	42.6%	456	42.0%	**576**	**42.3%**
Gender	Male	92	59.0%	26	49.1%	39	57.4%	629	63.3%	**786**	**57.7%**
	**Total**	**156**	**100.0%**	**53**	**100.0%**	**68**	**100.0%**	**1085**	**100.0%**	**1362**	**100.0%**
	No	138	88.5%	51	96.2%	56	82.4%	993	80.2%	**1238**	**90.9%**
Ventilation	Yes	18	11.5%	2	3.8%	12	17.6%	92	74.2%	**124**	**9.1%**
	**Total**	**156**	**100.0%**	**53**	**100.0%**	**68**	**100.0%**	**1085**	**100.0%**	**1362**	**100.0%**
	No	122	78.2%	46	86.8%	56	82.4%	939	80.7%	**1163**	**85.4%**
ICU admission	Yes	34	21.8%	7	13.2%	12	17.6%	146	73.4%	**199**	**14.6%**
	**Total**	**156**	**100.0%**	**53**	**100.0%**	**68**	**100.0%**	**1085**	**100.0%**	**1362**	**100.0%**
	No	66	42.3%	30	56.6%	49	72.1%	482	76.9%	**627**	**46.0%**
Comorbidities	Yes	90	57.7%	23	43.4%	19	27.9%	603	82.0%	**735**	**54.0%**
	**Total**	**156**	**100.0%**	**53**	**100.0%**	**68**	**100.0%**	**1085**	**100.0%**	**1362**	**100.0%**
	No	130	83.3%	50	94.3%	60	88.2%	950	79.8%	**1190**	**87.4%**
Asthma	Yes	26	16.7%	3	5.7%	8	11.8%	135	78.5%	**172**	**12.6%**
	**Total**	**156**	**100.0%**	**53**	**100.0%**	**68**	**100.0%**	**1085**	**100.0%**	**1362**	**100.0%**
	No	126	80.8%	44	83.0%	62	91.2%	817	77.9%	**1049**	**77.0%**
Diabetes	Yes	30	19.2%	9	17.0%	6	8.8%	268	85.6%	**313**	**23.0%**
	**Total**	**156**	**100.0%**	**53**	**100.0%**	**68**	**100.0%**	**1085**	**100.0%**	**1362**	**100.0%**
	No	124	79.5%	49	92.5%	61	89.7%	894	79.3%	**1128**	**82.8%**
Heart disease	Yes	32	20.5%	4	7.5%	7	10.3%	191	81.6%	**234**	**17.2%**
	**Total**	**156**	**100.0%**	**53**	**100.0%**	**68**	**100.0%**	**1085**	**100.0%**	**1362**	**100.0%**
	No	153	98.1%	48	90.6%	67	98.5%	1024	79.3%	**1292**	**94.9%**
Immune compromised	Yes	3	1.9%	5	9.4%	1	1.5%	61	87.1%	**70**	**5.1%**
	**Total**	**156**	**100.0%**	**53**	**100.0%**	**68**	**100.0%**	**1085**	**100.0%**	**1362**	**100.0%**
	No	148	94.9%	52	98.1%	65	95.6%	1035	79.6%	**1300**	**95.4%**
Chronic kidney disease	Yes	8	5.1%	1	1.9%	3	4.4%	50	80.6%	**62**	**4.6%**
	**Total**	**156**	**100.0%**	**53**	**100.0%**	**68**	**100.0%**	**1085**	**100.0%**	**1362**	**100.0%**
	No	154	98.7%	52	98.1%	67	98.5%	1053	79.4%	**1326**	**97.4%**
Neuromuscular dysfunction	Yes	2	1.3%	1	1.9%	1	1.5%	32	88.9%	**36**	**2.6%**
	**Total**	**156**	**100.0%**	**53**	**100.0%**	**68**	**100.0%**	**1085**	**100.0%**	**1362**	**100.0%**
	No	151	96.8%	53	100.0%	67	98.5%	1046	79.4%	**1317**	**96.7%**
Chronic hematological disorder	Yes	5	3.2%	0	0.0%	1	1.5%	39	86.7%	**45**	**3.3%**
	**Total**	**156**	**100.0%**	**53**	**100.0%**	**68**	**100.0%**	**1085**	**100.0%**	**1362**	**100.0%**
	No	142	91.0%	51	96.2%	63	92.6%	1019	79.9%	**1275**	**93.6%**
Chronic lung disease	Yes	14	9.0%	2	3.8%	5	7.4%	66	75.9%	**87**	**6.4%**
	**Total**	**156**	**100.0%**	**53**	**100.0%**	**68**	**100.0%**	**1085**	**100.0%**	**1362**	**100.0%**
	No	156	100.0%	53	100.0%	68	100.0%	1075	79.5%	**1352**	**99.3%**
Chronic liver disease	Yes	0	0.0%	0	0.0%	0	0.0%	10	100.0%	**10**	**0.7%**
	**Total**	**156**	**100.0%**	**53**	**100.0%**	**68**	**100.0%**	**1085**	**100.0%**	**1362**	**100.0%**

* Others: SARS-CoV-2, adenovirus, rhinovirus, negative results.
